# Comparison of Therapeutic Approaches to Addicted Patients with Central Sleep Apnea

**Published:** 2018-03

**Authors:** Christoph Schoebel, Amir Ghaderi, Babak Amra, Forogh Soltaninejad, Thomas Penzel, Ingo Fietze

**Affiliations:** 1 Department of Cardiology and Angiology, Center of Sleep Medicine, Charité – Universitätsmedizin Berlin, Berlin, Germany; 2 Department of Internal Medicine, Isfahan University of Medical Sciences, Isfahan, Iran; 3 Pulmonary Ward, Bamdad Respiratory and Sleep Research Center, Isfahan University of Medical Sciences, Isfahan, Iran; 4 Pulmonary Ward, Isfahan University of Medical Sciences, Bamdad Respiratory and Sleep Research Center, Isfahan, Iran; 5 Center of Sleep Medicine, Charité –Universitätsmedizin Berlin, Berlin, Germany; 6 Department of Cardiology and Pulmonology, Center of Sleep Medicine, Charité – Universitätsmedizin Berlin, Berlin, Germany

**Keywords:** Central sleep apnea, Continuous positive airway pressure, Bi-level positive airway pressure, Supplemental O_2_, Addiction

## Abstract

**Background:**

Nowadays, the most practical approaches used to treat sleep apnea, are Continuous Positive Airway Pressure (CPAP), Bi-level Positive Airway Pressure therapy (BPAP), supplemental O_2_, servoventilation and/or a combination of these approaches simultaneously. However, each leads to different consequences in opioid related Central Sleep Apnea (CSA) patients. Given the high prevalence of CSA and frequently use of opioids worldwide, it seems that evaluation of the condition in these patients is required to determine their responsiveness to the above mentioned treatments and to choose the most appropriate therapy.

**Materials and Methods:**

This longitudinal cross-sectional study included 41 opioid related CSA patients who underwent a step-by-step protocol (including CPAP, CPAP + O_2_ and BPAP) in which if the patient was nonresponsive to a treatment, the next therapy was applied. If the patient was nonresponsive to all of these approaches, only oxygen was administered. Finally, the collected data were analyzed with SPSS software (ver. 22).

**Results:**

Among 41 participants, the responsiveness to CPAP, CPAP+O_2_ and BPAP were 41.5%, 14.6% and 39%, respectively versus 4.9% nonresponsive patients to all above mentioned therapies. In patients with CSA and opium addiction, the CPAP and BPAP were the most effective treatments. In this group of patients, better response in the presence of higher Apnea–Hypopnea Index (AHI) was observed to BPAP, whereas better response in patients with lower AHI was to CPAP+O_2_

**Conclusion:**

Accordingly, CPAP and BPAP are successful approaches to treat opioid related CSA patients in various medical conditions including long-run addiction course, concurrent smoking and addiction but it appears that further studies are essential.

## INTRODUCTION

The Sleep-Disordered Breathing (SDB) which is generally classified into Obstructive Sleep Apnea (OSA) and Central Sleep Apnea (CSA), has been reported in 6.5 to 9 percent of adults (1).

Despite the lower prevalence of CSA rather than OSA (2), CSA syndromes can be categorized into five subsets: primary (idiopathic) CSA, CSA due to Cheyne–Stokes breathing, CSA due to medical disorder without Cheyne–Stokes breathing, high-altitude periodic breathing and CSA due to drug or substance (3,4).

To verify this categorization, some studies indicated increased risk of OSA due to opioids (5, 6). For examples 75% of opioid users had experienced more than 5 apneic episodes per hour of sleep (7) and/or 30% of cancer patients using opioids to manage the pain, were dealt with increased CSA episodes (8).

Given chronic opioid users response to hypercapnia through a depressed ventilation versus their augmented response to hypoxia, the lack of regulation about respiratory chemical reactions may lead to an instable breathing (9,7, 10). Hence, some studies have pointed out that sleep apneas could be the cause of mortality induced by over-use of opioids (11,12). However, unfortunately CSA treatment in opioids addicts is rarely investigated (4,13).

Taking into account the concerns about uncontrollable pain and/or increased desire to use opioids, perhaps decreased dosage of opioids cannot be considered as an easy solution to treat (14). Applied treatment options from 2007 so far, including Positive Airway Pressure (PAP) (14), servoventilation (ASV) (15,16), Continuous Positive Airway Pressure (CPAP) (7), BPAP+O_2_ or CPAP+O_2_, as a single therapy or step-by-step were used to achieve the desired response (17), although Farney et al. reported no improvement in ataxic breathing and overnight oxygenation in addicted CSA patients using ASV (18).

Thus, given the purity of evidence in literature evaluating the efficacy of available CSA therapies, the lack of similarity of these therapies can be anticipated (19,20).

The contradictory results can be attributed to different definitions of CSA, therapeutic conditions and procedures as well as opioids dosage and medical conditions (4).

Given increasing opioid use worldwide, the role of etiologies as well as concomitants in therapy choice and the lack of a global standard treatment; the current study aimed to evaluate some therapy options in CSA with respect to the histories of concomitants and opioid use in the patients.

## MATERIALS AND METHODS

This longitudinal cross-sectional study aimed to compare therapeutic approaches to CSA in addicts. Taking into account the low prevalence of CSA and accordingly the likelihood of small available sample, initially all 41 addicted male patients presented at the centers of sleep and breathing disorders in Isfahan during April 2017 to March 2018 were included by census.

Using standard Polysomnography (PSG) and/or during two-sectional diagnostic PSG for hours, these patients were diagnosed when five or more central apneas and/or central hypopneas are present per hour.

The demographic and clinical characteristics in CSA and hypoxia including age, sex, weight, height, Body Mass Index (BMI), blood pressure, neck circumference, course of using opium, apnea-hypopnea index, Central Apnea Index (CAI), use of opioid and smoking were recorded in an encoded form to keep the privacy (ethical issues).

When CSA patient presented at the center, the patient underwent positive air pressure titration from a low pressure to the pressure of 15 cmH_2_O. If the patient was nonresponsive to CPAP, Bi-level Positive Airway Pressure (BPAP) was used. It should be noted that CPAP and BPAP titrations were performed based on the American Academy of Sleep Medicine (AASM 2008) guideline for adults (21).

Afterwards, providing a CAI of higher than 5 with the oxygen saturation of lower than 93 percent, was considered as nonresponsive case to therapeutic approaches and for the patient, only oxygen was administered and discharged.

Finally the collected data were analyzed with SPSS (ver. 22). The qualitative data were reported by frequencies (percentage) and the quantitative data were expressed as Mean ± SD. Also, to compare quantitative data of each therapeutic approach with others and the response to treatment two by two, the exact Fisher test was applied and to compare all four therapeutic approaches existing in the study protocol, the Chi-square test was used. Given that the Kolmogorov–Smirnov test indicated the normality of data distribution, for quantitative data and in order to compare the mean variables between responsive and nonresponsive patients to the treatment, we used Independent samples t-test. To compare mean variables of four therapeutic approaches, one-way ANOVA was used and the two by two comparison of therapeutic approaches was made by the post hoc Tukey test. In all analyses, we used a significance level of < 0.05.

## RESULTS

This study included 41 male addicted patients with CSA [mean age=63.78±11.95 years; mean Apnea–Hypopnea Index (AHI)=32.97±21.24/h]. Among these patients, those with diabetes mellitus, hypertension and hyperlipidemia (HLP) were 48.8, 36.6 and 17.1%, respectively of which 50% were not only opioid addicts but also smokers (Table 1).

**Table 1.** Baseline and clinical characteristics of patients with CSA

**Characteristics**		N(%)^†^ or Mean(SD)^*^
**Age; year**		63.78(11.95)^*^
**Sex; Male**		41(100.0)^†^
**BMI; kg/m^2^**		31.02(6.94)^*^
**Height; cm**		168.88(9.86)^*^
**Weight; kg**		81.22(17.62)^*^
**Neck circumference; cm**		38.96(2.13)^*^
		32.97(21.24)^*^
**AHI; no./h^‡^**	**Mild CSA**	15(36.6)^†^
	**Moderate CSA**	15(36.6)^†^
	**Severe CSA**	11(26.8)^†^
**Clinical records**		
	**DM**	20(48.8)^†^
	**HTN**	15(36.6)^†^
	**HLP**	7(17.1)^†^
**Smoking**		23(56.1)^†^
**Addiction course; year**		10.76±2.74

‡: AHI group: Mild CSA (5≤AHI< 15 events/h); moderate CSA (15≤AHI< 30 events/h); and severe CSA (AHI ≥ 30 events/h).

As shown, 17 patients were responsive to CPAP versus 24 nonresponsive patients. There were significant difference between these patients in mean AHI and smoking (P value<0.05). The majority of nonsmoking addicts with rather high degree of AHI were more responsive to CPAP. Moreover, the addiction course among these CPAP responders (mean addiction course=9.71±2.64y) was significantly shorter than nonresponsive patients to CPAP (mean addiction course=11.50±2.62y) (P value=0.038) (Table 2).

**Table 2.** Demographics and baseline characteristics of CPAP responders vs. non-responders

**Characteristics**		**Response (n=17)**	**Non-Response (n=24)**	**P value**
**Age; year**		67.18±12.25	60.50±11.17	0.078
**BMI; kg/m^2^**		29.00±5.03	28.27±5.15	0.652
**Height; cm**		170.47±5.76	167.75±11.95	0.391
**Weight; kg**		82.18±14.38	80.54±19.86	0.774
**Neck circumference; cm**		41.63±2.83	40.08±2.27	0.067
**AHI; no./h^‡^**		38.85±10.54	28.06±19.98	0.041
**Clinical records**				
	**DM**	10/20(50)	10/20(50)	0.350
	**HTN**	7/15(46.7)	8/15(53.3)	0.745
	**HLP**	4/7(57.1)	3/7(42.9)	0.421
**Smoking**		5/23(21.7)	18/23(78.3)	0.005
**Addiction course; year**		9.71±2.64	11.50±2.62	0.038

Data shown n/N(%) or Mean ± SD

Furthermore, among 24 CPAP nonresponsive patients, 6 patients were responsive to CPAP+O_2_, versus 18 nonresponsive patients. A lower mean AHI was reported for CPAP+O_2_ responders (21.67±15.87 vs. 34.33±12.17; P value=0.045) (Table 3).

**Table 3.** Demographics and baseline characteristics of CPAP+O_2_ responders vs. non-responders

**Characteristics**		**Response (n=6)**	**Non-Response (n=18)**	**P value**
**Age; year**		57.68±5.99	61.44±12.43	0.485
**BMI; kg/m^2^**		28.78±5.75	28.10±5.11	0.785
**Height; cm**		169.50±3.89	167.17±13.68	0.688
**Weight; kg**		82.67±16.95	79.83±21.15	0.770
**Neck circumference; cm**		41.42±2.05	38.78±2.10	0.088
**AHI; no./h^‡^**		21.67±15.87	34.33±12.17	0.045
**Clinical records**				
	**DM**	4/10(40.0)	6/10(60.0)	0.192
	**HTN**	3/8(37.5)	5/8(62.5)	0.362
	**HLP**	0/3(0)	3/3(100)	0.285
**Smoking**		5/18(27.8)	13/18(72.2)	0.899
**Addiction course; year**		11.50±2.42	11.48±2.75	0.953

‡: AHI group: Mild CSA (5≤AHI< 15 events/h); moderate CSA (15≤AHI< 30 events/h); and severe CSA (AHI ≥ 30 events/h), Data shown n/N(%) or Mean ± SD

On the other hand, among 18 nonresponsive patients to CPAP+O_2_, 16 patients were responsive to BPAP versus 2 nonresponsive patients. There was no difference between responsive and nonresponsive patients to treatment in terms of basic and clinical factors (P value>0.05) (Table 4).

**Table 4.** Demographics and baseline characteristics of BPAP responders vs. non-responders

**Characteristics**		**Response (n=16)**	**Non-Response (n=2)**	**P value**
**Age; year**		61.19±12.64	63.50±14.85	0.813
**BMI; kg/m^2^**		28.29±5.41	26.60±1.21	0.673
**Height; cm**		167.06±14.56	168.00±3.12	0.930
**Weight; kg**		80.44±22.44	75.00±21.08	0.743
**Neck circumference; cm**		41.72±2.34	39.63±2.14	0.249
**AHI; no./h^‡^**		35.24±21.05	28.67±12.87	0.677
**Clinical records**				
	**DM**	6/6(100)	0/6(0)	0.529
	**HTN**	5/5(100)	0/5(0)	0.510
	**HLP**	3/3(100)	0/3(0)	0.686
**Smoking**		11/13(84.6)	2/13(15.4)	0.352
**Addiction course; year**		11.69±2.77	10.00±2.83	0.430

Data shown n/N(%) or Mean ± SD

Ultimately, the evaluation of basic and clinical characteristics in association with responsiveness to protocol demonstrated that nonresponsive patients to the above mentioned therapies who were discharged following oxygen administration, were older than CPAP+O_2_ and BPAP responders. Also, a lower mean BMI was reported for these nonresponsive patients, although the difference was not statistically significant (P value>0.05). On the other hand, mean age of CPAP responders was significantly higher than CPAP+O_2_ responders (67.18±12.25 vs. 57.68±5.99; P value=0.038). In addition, CPAP and BPAP responders showed the severest CSAs (mean AHI; 38.85±10.54 no. /h and 35.24±21.05 no. /h) compared to CAPAP+O_2_ responders with mildest CSA (mean AHI=21.67±15.87 no. /h) (P value<0.05). Also, a considerable percentage of smoking opioid addicts had shown a desirable response to BPAP with the longest course of using opioids (mean=11.69±2.77y) versus CPAP responders with the shortest course of using opioids (9.71±2.64y) (P value<0.05) (Table 5).

**Table 5.** Demographics and baseline characteristics of individuals with response to the protocol

**Characteristics**	**CPAP (n=17)**	**CPAP+O_2_ (n=6)**	**BPAP (n=16)**	**O_2_ (n=2)**
**Age; year**	67.18±12.25^†^	57.68±5.99^*^	61.19±12.64	63.50±14.85
**BMI; kg/m^2^**	29.00±5.03	28.78±5.75	28.29±5.41	26.60±1.21
**Height; cm**	170.47±5.76	169.50±3.89	167.06±14.56	168.00±3.12
**Weight; kg**	82.18±14.38	82.67±16.95	80.44±22.44	75.00±21.08
**Neck circumference; cm**	41.63±2.83	41.42±2.05	41.72±2.34	39.63±2.14
**AHI; no./h^‡^**	38.85±10.54^†^	21.67±15.87^*#^	35.24±21.05^†^	28.67±12.87
**Clinical records**				
**DM(n=20)**	10/20(50.0)	4/20(20.0)	6/20(30.0)	0/20(0)
**HTN(n=15)**	7/15(46.7)	3/15(20.0)	5/15(33.3)	0/15(0)
**HLP(n=7)**	4/7(57.1)	0/7(0)	3/7(42.9)	0/7(0)
**Smoking (n=23)**	5/23(21.7)^†¶^	5/23(21.7)^*^	11/23(43.8)^†^	2/2(8.8)^*^
**Addiction course; year**	9.71±2.64^#^	11.50±2.42	11.69±2.77^*^	10.00±2.83

Data shown n/N(%) or Mean ± SD

*: Significant level of comparison vs. CPAP

†: Significant level of comparison vs. CPAP+O_2_

#: Significant level of comparison vs. BPAP

¶: Significant level of comparison vs. O_2_

## DISCUSSION

According to the results, all patients with CSA were males with the mean age of 63.78±11.95 years. Moreover, more than 50 percent of these patients were smokers with the addiction course of 10.76±2.74 years.

In this regard, the prevalence of CSA is considerably higher in men and it increases with age. For example a community cohort of 741 men showed that the CSA prevalence was estimated to be 0.4% overall among those aged 65 and older (22). Also, afterwards another cohort of 2,911 men reported that the CSA prevalence (CAI ≥ 5) was higher among those aged 65 and older (23). Many studies have reported that opioid use is common in CSA patients and these patients may be at risk of death, whereas their mortality rate has been estimated to be 3% (24).

The findings of therapeutic approaches indicated that among 41 patients, responsive patients to CPAP, CPAP + O_2_ and BPAP were 41.5%, 14.6% and 39%, respectively. At the end, 4.9% of patients remained nonresponsive to these three approaches and thus, this group of patients underwent oxygen administration and was discharged.

Paying attention to addicted patients with CSA is rarely investigated. The primary report about treating this group of CSA patients with PAP, backs to 2007 (16). Also, in 2008 and 2009, using PAP as a therapeutic approach for chronic opioids users with CSA was successful (15,25). In 2012, two larger studies were conducted to treat CSA as well (17,26). The results of these two studies suggested success of using ASV to treat CSA in association with opioids in 59.6% of cases (26). Another study including 151 patients with CSA, of which 41 patients were opioid addicts, showed that the success of CPAP, CPAP + O_2_ and BPAP + O_2_ based on a step-by-step protocol were 54%, 27% and 10%, respectively; thus, using CPAP as the primary choice and CPAP+O_2_ as the second choice for treatment of this group of patients were recognized (17).

On the other hand, the assessment of patients' condition impact on responsiveness to therapeutic approaches in the current study showed that the majority of CPAP responders were not smokers but with a rather high AHI. Also, this therapy for patients with longer addiction course was unsuccessful. Then, using CPAP+O_2_ was successful only for patients with a lower AHI, while addiction course could not play a determinant role in their responsiveness. Finally, of 18 remained patients, 16 cases were responsive to BPAP versus two nonresponsive patients who were discharged after oxygen administration. There was no difference between these patients in terms of basic and clinical factors. It showed be noted that the comparison of basic and clinical characteristics in responsive patients to the step-by-step protocol (including CPAP, CPAP+O_2_ and BPAP) suggested a more desirable responsiveness to both approaches of CPAP and BPAP in addicted CSA patients (with AHI>25 no. /h) compared to CPAP+O_2_ (with AHI>15).

In this regard, many studies have supported the effect of CPAP on CSA (17,24,27,28).

For example, Dohi et al. found that 11 patient (with AHI<15) showed a significant improved AHI, while only a slightly improved AHI was reported in 9 patients with high AHI (AHI≥15). In the latter group, a significant improved AHI was reported using BPAP titration. Also, longer mean duration of Cheyne-Stokes respiration (CSR) and higher plasma Brain Natriuretic Peptide (BNP) as well as significantly lower PaCO_2_ and fewer obstructive episodes were reported in nonresponsive patients to CPAP than CPAP responders (29).

Also, another study reported an improved AHI (AHI<10) in 28 patients (59.6%) using ASV for opioid-related CSA treatment. Using the criteria of AHI<5 under optimal ASV end-expiratory pressure, a similar result was reported in 29 patients (61.7%) (26).

Actually the CPAP therapy could result in restoration of upper airway patency as well as stabilization of the respiratory control system. CSA is defined as cycles of apnea and hyperpnea, which induces hypocapnea. CPAP dampens hyperventilation and stabilizes respiration (17).

In the past, few studies with contradictory results were done with the aim of comparing the efficacies of various treatments in addicts with CSA. The different results could be achieved due to different definitions of CSA, therapeutic approaches and medical conditions as well as the type of opioids and the course of opioid use (4). The CSA severity in opioid users could be varied depending on the opioid dosage and the course of use (14). In this respect, Troitino et al. compared the treatment outcome and adherence rate to CPAP, BPAP and ASV in 34 opioid-related CSA (O-CSA) patients and 61 idiopathic CSA (I-CSA) patients. In their study, CSA was defined as CAI>5 per hour and CAI≥50% of the AHI. The findings suggested CPAP, BPAP, and ASV as the alternative choices to eliminate CSA and to improve oxygenations in both CSA groups. The initial CPAP compliances of 24 and 38% were reported for O-CSA and I-CSA groups, respectively. A similarly low PAP adherence rate (~20 %) was reported for both groups of O-CSA and I-CSA during 12 months (24).

A 12-months follow-up to assess the adherence rate, could be considerable although a long-run follow-up was not performed in the current study.

Perhaps the reduction in CAI and arousal index with PAPs was associated with a reduction in loop gain, hyperventilation and finally cyclic periodic breathing pattern (30).

One group of the potent respiratory depressants are opioids which may lead to hypoxia and hypercapnia in some opioid related CSA patients. It appears that to improve oxygenation and to reduce CSA events in the majority of the opioid-related CSA patients, using PAP titration can be beneficial. However, dealing with low PAP adherence is challenging (9).

Finally, according to the results, the concurrence of addiction and smoking and/or longer course of addiction resulted in significant increased success rate of BPAP versus CPAP.

Some studies investigating the relationship between opioids use and CSA, displayed that there is a dependent correlation between opioid dosage and CSA and opioids use can reduce slow wave as well as REM sleep stages (31,32).

In the literature it has been reported that CPAP without oxygen supplement was ineffective in three patients with a long-run use of opioids and only it can slightly prevent hypoxemia (33).

Also, some studies have pointed out that ASV and BPAP are more expensive than CPAP and in some cases without desired response. Thus, the practical approach can be still using CPAP as the primary therapy, although ASV and BPAP can be applied as alternatives if CPAP failed (9,25, 26). Given the various presentations of CSA and its association with opioids use and variations in reactions to opioids (7,34), using a medical approach with paying attention to individual characteristics (e.g. overnight hypoxemia, CSA, awaking reactions, daytime sleepiness/consciousness and so on) can be more rational and safer approach.

Therefore, further RTC studies with larger sample size are recommended to compare basic and clinical outcomes of CPAP and other therapies. Furthermore, lack of long-run patients' follow-up after treatment is one of the limitations of this study. Thus, it is suggested to follow-up patients for a longer period of time in further studies with a similar staged protocol and conduct independent studies to evaluate two-by-two these approaches to provide more appropriate documented results using larger sample size.
